# Clinical outcomes in patients with solid tumors living in rural and urban areas followed via telemedicine: experience in a highly complex latin american hospital

**DOI:** 10.1186/s12885-023-10717-5

**Published:** 2023-03-16

**Authors:** Juan Guillermo Restrepo, Juliana Alarcón, Andrés Hernández, Saveria Sangiovanni, Sofía González, Kelly Gallego, Evelyn E. Peña-Zárate, Laura Libreros-Peña, María Fernanda Escobar

**Affiliations:** 1 Departament of Hematology-Oncology, Fundación Valle del Lili, Cali, Colombia; 2 Centro de Investigaciones Clínicas, Fundación Valle Lili, Cali, Colombia; 3 Facultad de Ciencias de la Salud, Universidad Icesi, Cali, Colombia; 4 Department of Gynecology and Obstetrics, Universidad Icesi, Cali, Colombia; 5 Department of Telemedicine, Fundación Valle del Lili, Cra 98 Nro.18-49, Cali, 7600.2 Colombia

**Keywords:** Oncological patients, Solid tumors, Telehealth, Telemedicine, Latin America

## Abstract

**Background:**

Difficulties in cancer services access increase the burden of disease and mortality in rural areas, and telehealth can be a useful tool to address these inequalities.

**Objective:**

We aimed to describe the outcomes of patients in rural and urban areas with solid tumors managed by oncologists through telemedicine.

**Methods:**

We conducted a retrospective cohort study of patients with solid tumors from March to December 2020. A total of 1270 subjects with solid tumors were included, 704 living in urban areas and 566 in rural areas.

**Results:**

The most frequent tumors were breast (51.8%) and prostate (12.4%). The trend of telemedicine care was similar for both populations; in-person care was more frequent in the urban population. There were no differences in referral to the emergency room, need for hospitalization, and mortality for both groups.

**Conclusion:**

Telemedicine is a care modality that reduces barriers in the care of patients with solid tumors, evidencing similar outcomes regardless of living in rural or urban areas.

## Introduction

Cancer is one of the leading causes of death worldwide, representing almost one in six deaths, and given its high burden, it is considered a public health concern, and a major challenge in low- and middle-income countries [1]. In Colombia, the incidence of cancer in 2020 was 182.3 cases per 100,000 population, with prostate, breast, and colon-rectum cancers being the most frequent. For the same year, the mortality rate was 84.7 per 100,000 cases [2].

The rural population faces multiple health inequalities, motivated by the lack of health policies and structural, socioeconomic, and environmental barriers [3]. In cancer, health disparities vary from prevention, treatment, and follow-up, which are generally related to living conditions, work, medical insurance, economic income, educational level, and area of residence [4]. In Colombia, it is estimated that the rural population represents 23–24% of the total population [5]. Nevertheless, approximately 85% of the oncological services available are located in urban areas, and not all of them provide basic services for cancer treatment (chemotherapy, radiotherapy, and surgery) [6].

The COVID-19 pandemic has increased the barriers to access to health care services worldwide, being more pronounced in rural populations with unfavorable socioeconomic conditions and delaying timely disease diagnosis and treatment [7]. Consequently, different strategies based on telehealth have significantly increased its use and implementation to improve those limitations [8]. These technological resources in cancer can be applied in different scenarios [9], such as first-time care or control by a specialist, postoperative follow-up [10], palliative care [11], symptom management, and remote chemotherapy supervision, among others, providing high levels of patient and family satisfaction [9].

There is limited research and interventions involving telehealth in the follow-up of cancer patients in low- and middle-income countries. The present study aims to describe our clinical experience and clinical outcomes of patients with solid tumors living in urban and rural areas who received care through a telemedicine program implemented in a high complexity Latin American hospital.

## Methods

### Design

We conducted a retrospective cohort study of adult patients (≥ 18 years old) diagnosed with any type of solid tumor who required at least one consultation with an oncologist specialist via telemedicine from March to December 2020 at Fundación Valle del Lili. Patients whose current clinical condition required in-person care for vital signs or physical assessment were excluded.

The Institutional Review Board at Fundación Valle del Lili approved the protocol of this study (IRB/EC Protocol number 101–2021). Informed consent was waived by the institutional review board of Fundación Valle del Lili, as it was classified as risk-free according to national resolution (No. 008430 of 1993, article 11, numeral A) of the Ministry of Health and Social Protection of Colombia.

### “Siempre” telemedicine program

Prompted by the mandatory lockdown measures, Fundación Valle del Lili (FVL) implemented an outpatient telemedicine service called “Siempre”, which means “always” in English, as an outpatient care strategy to continue delivering care to patients. FVL is a high-complexity university hospital located in the city of Santiago de Cali, Colombia, and a referral center that provides care to approximately 12,700 patients annually, mostly inhabitants of the southwestern region of the country [12].

This model of attention uses the Microsoft Teams® platform to establish real-time video calls between the oncologist and the patient. Throughout medical attention, the oncologist updates the patient’s clinical status, asks about the presence of symptoms or alarm signs, the need for emergency room visits or hospitalizations since the last appointment, and analyzes the latest laboratory or imaging results requested. All this information was recorded in the institutional electronic medical record system (SAP), through which requests for procedures, lab or imaging tests, referral for evaluation by other necessary specialties, and formulation of medications were made. After the service was completed, the documents were sent in PDF format to the patient’s e-mail address.

### Variables and data collection

All data were retrospectively obtained from the institutional electronic medical records and recorded in the BdClinic database software. We included sociodemographic, clinical, and follow-up characteristics such as age, sex, job occupation, health insurance, type of solid tumor diagnosis, management, current disease status, and clinical outcomes.

### Statistical analysis

Once the data had been collected, quality control was performed by comparing a random sample of 10% with the information recorded in the medical records, without inconsistencies. An exploratory analysis was performed to detect missing data and extreme values, followed by a descriptive analysis of the data.

Qualitative variables were summarized through absolute frequencies and percentages; measures of central tendency and dispersion were employed according to the obtained distribution through the Shapiro‒Wilk test. An evaluation using a Chi-square association test was performed, considering a statistical significance of 0.05. Analysis was performed using StataCorp. 2015. *Stata Statistical Software: Release 14*. College Station, TX: StataCorp LP.

## Results

During the 9 months in which the study was carried out, 2061 patients were attended to by the oncology specialty telemedicine service. Of these patients,1270 were diagnosed with solid tumors; 704 lived in urban areas, and 566 lived in rural areas. The median age was 66 years (IQR 55–75), 70.9% were women, and 69.7% had some comorbidity, with more frequent hypertension and diabetes mellitus. The most frequent solid tumors were breast (53.6%), prostate (12.1%), colon and rectum (8.3%) (Table 1).

**Table 1:** Sociodemographic characteristics by area of residence

Characteristics	Urban Area n = 704	Rural Area n = 566	Total n = 1270
Age, year *	65 (54–75)	66 (57–75)	66 (55–75)
**Gender**			
Female	495 (70.3%)	406 (71.7%)	901 (70.9%)
Male	209 (29.7%)	160 (28.3%)	369 (29.1%)
**Comorbidities** ^**a**^	482 (68.5%)	371 (65.5%)	853 (69.7%)
Arterial hypertension	271 (38.5%)	248 (43.8%)	519 (42.4%)
Diabetes Mellitus	95 (13.5%)	89 (15.7%)	184 (15.0%)
Chronic kidney disease	42 (6.0%)	12 (2.1%)	54 (4.4%)
Coronary heart disease	45 (6.4%)	18 (3.2%)	63 (5.1%)
Respiratory system disease	24 (3.4%)	24 (4.2%)	48 (3.9%)
**Tumor type**			
Breast	365 (51.8%)	316 (55.8%)	681 (53.6%)
Colon and rectum	63(8.9%)	42 (7.4%)	105 (8.3%)
Lung	24 (3.4%)	23 (4.1%)	47 (3.7%)
Prostate	87 (12.4%)	67 (11.8%)	154 (12.1%)
Esophagus/Gastric	12 (1.7%)	23 (4.1%)	35 (2.8%)
Head/Neck	22 (3.1%)	20 (3.5%)	42 (3.3%)
Melanoma	6 (0.9%)	7 (1.2%)	13 (1.0%)
Other	125 (17.8%)	68 (12.0%)	193 (15.2%)

* Median (IQR)

^a^ A patient may have one or more of the following diseases: arterial hypertension, diabetes mellitus, chronic kidney disease, coronary heart disease, chronic diseases of the respiratory system, and other comorbidities

**Fig. 1:**
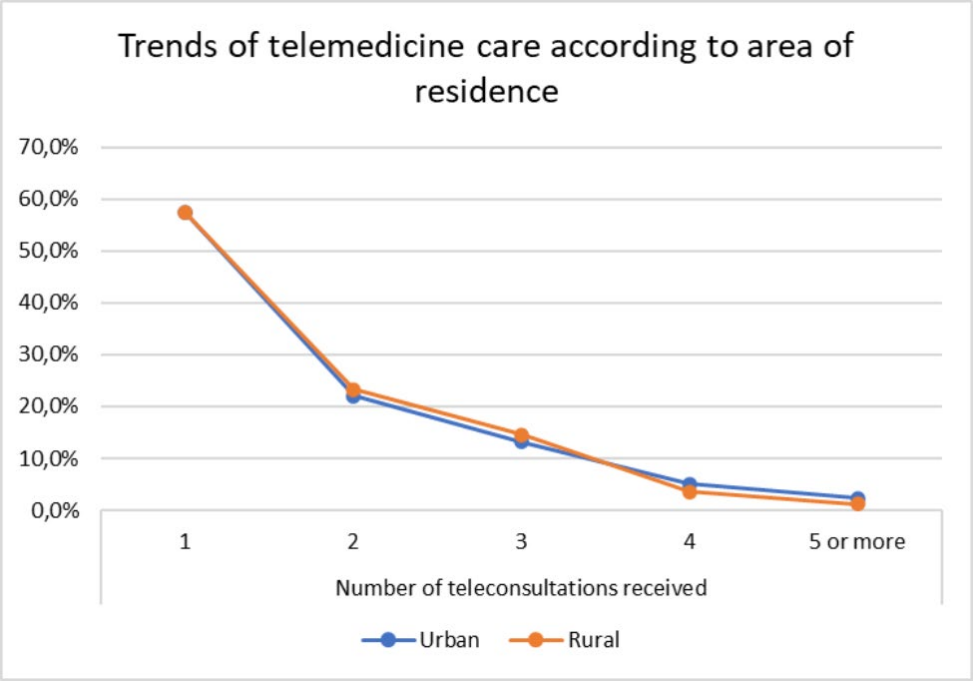
Trends of telemedicine care according to area of residence

**Fig. 2:**
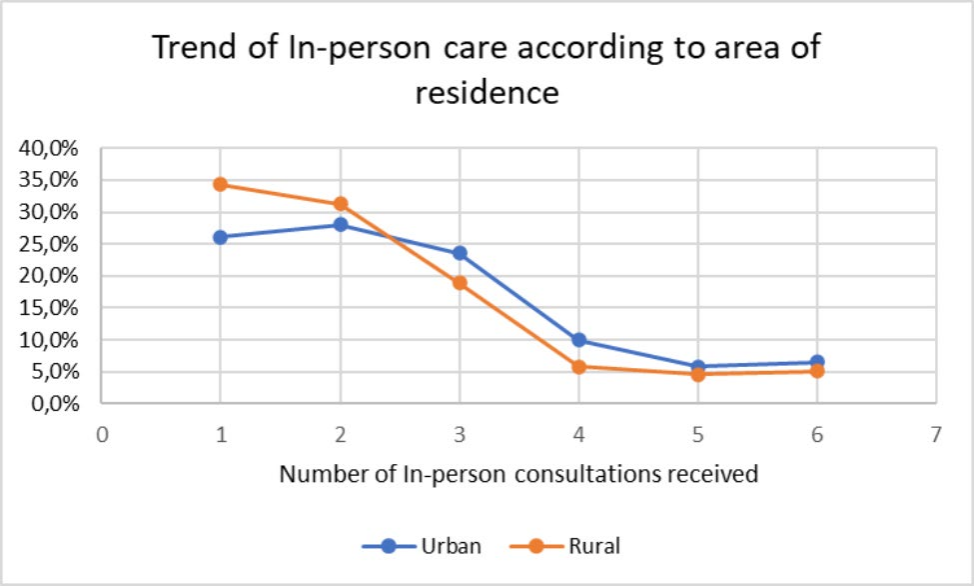
Trend of in-person consultations according to area of residence

**Fig. 3:**
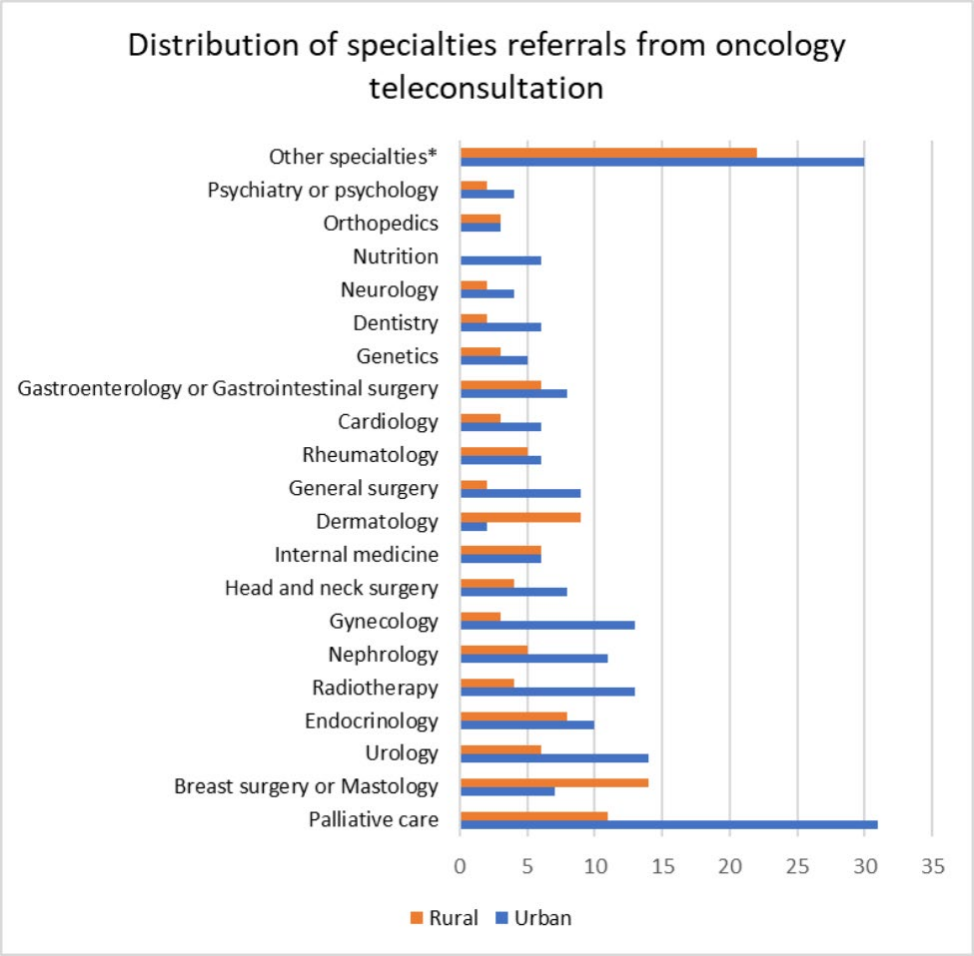
Distribution of specialty referrals from oncology telemedicine attention. (* Other specialty categories include colon and rectum surgery, hepatology, pneumology, vascular surgery, ophthalmology, thoracic surgery, pain clinic, physiatry, maxillofacial surgery, oncologic surgery, infectiology, neurosurgery, proctology, geriatrics, hematology, and otorhinolaryngology)

Most of the patients received at least 1 telemedicine consultation (urban 57.39% vs. rural 57.42%), 40.8% received 2 to 4 telemedicine consultations (urban 40.34% vs. rural 41.34%), and 1.81% received 5 or more telemedicine consultations (urban 2.27% vs. rural 1.24%) (Fig. 1). The percentage of in-person consultations was higher in the urban group, except for those who received only one in-person consultation, which was higher in the rural group; 29.7% of the patients did not receive care by this modality (urban 26.14% vs. rural 34.28%) (Figs. 2 and 3).

Among the clinical characteristics and oncological status, 72.6% of the patients were in a local stage of cancer without metastasis (urban 71.6% vs. rural 73.9%, p = 0.4); 63.1% were currently under treatment (urban 61.9% vs. rural 64.7%, p = 0.4), and of these patients, 55.6% had maintenance intention (urban 54.1% vs. rural 57.3%, p = 0.042). The functional state evaluated using the ECOG scale was as follows: 69.0% ECOG 0, 19.2% ECOG 1, 5.5% ECOG 2, and 6.3% ECOG 3–4, without differences between groups (Table 2).

**Table 2:** Clinical characteristics and oncological status by area of residence

Characteristics	Urban Area n = 704	Rural Area n = 566	Total n = 1270
Time since cancer diagnosis, months*	34.0 (16.0–60.0)	36.0 (18.0–60.0)	36.0 (17.0–60.0)
**Cancer status**			
Local	504 (71.6%)	418 (73.9%)	922 (72.6%)
Metastatic	200 (28.4%)	148 (26.1%)	348 (27.4%)
**Cancer therapy**			
Currently in treatment	436 (61.9%)	366 (64.7%)	802 (63.1%)
Recently^a^	61 (8.7%)	47 (8.3%)	108 (8.5%)
Previously^b^	180 (25.6%)	140 (24.7%)	320 (25.2%)
Has not been treated	27 (3.8%)	13 (2.3%)	40 (3.1%)
**Treatment intent** ^**c**^			
Curative/Adjuvant	185 (42.4%)	131 (35.8%)	316 (39.4%)
Palliative	15 (3.4%)	25 (6.8%)	40 (5.0%)
Maintenance	236 (54.1%)	210 (57.3%)	446 (55.6%)
**Functional status - ECOG scale**			
0 - Fully active, able to carry on all predisease performance without restriction	262 (69.3%)	230 (69.7%)	492 (69.0%)
1 - Restricted in physically strenuous activity but ambulatory and able to carry out work of a light or sedentary nature, e.g., light housework, office work	73 (19.3%)	64 (19.1%)	137 (19.2%)
2 - Ambulatory and capable of all selfcare but unable to carry out any work activities; up and about for more than 50% of waking hours	20 (5.3%)	19 (5.7%)	39 (5.5%)
3 - Capable of only limited selfcare; confined to a bed or chair for more than 50% of waking hours	18 (4.8%)	14 (4.2%)	32 (4.5%)
4 - Completely disabled; cannot perform any selfcare; totally confined to a bed or chair	5 (1.3%)	8 (2.4%)	13 (1.8%)
No data	505	52	557

* Median (IQR)

^a^ Patients who completed treatment 6 months before the start of the study

^b^ Patients who completed treatment more than 6 months before the start of the study

^c^ Only patients with active treatment during the study period were considered

ECOG: Eastern Cooperative Oncology Group

Regarding telemedicine attention, 109 patients had alterations at the physical exam, and 220 patients had laboratory test alterations, mainly in the blood count (15.5%) and creatinine (10.1%); 42.3% of the patients required diagnostic images, without a difference between groups (Table 3). Moreover, 25.3% of the patients were referred to other specialists, with a significant difference between areas of residence (Table 3). The most common specialties were palliative care, breast surgery, and urology (Fig. 4).

**Table 3:** Oncology Appointment Care Characteristics

Characteristics	Urban Area n = 704	Rural Area n = 566	Total n = 1270	p value
Alterations detected at physical exam*	63 (8.5%)	46 (9.5%)	109 (8.6%)	0.2
Laboratory test alterations at consultation*				
Bilirrubin	9 (4.3%)	3 (1.9%)	12 (3.3%)	0.3
Transaminases	23 (8.0%)	16 (6.9%)	39 (7.5%)	0.8
Creatinine	32 (10.8%)	22 (9.2%)	54 (10.1%)	0.6
Alkaline phosphatase	21 (8.3%)	12 (6.4%)	33 (7.5%)	0.6
Blood count	49 (15.5%)	39 (15.4%)	88 (15.5%)	0.9
Request for diagnostic imaging in the consultation*	269 (42.0%)	241 (42.6%)	537 (42.3%)	0.9
Referral to other specialties in the consultation*	202 (28.7%)	120 (21.2%)	322 (25.3%)	0.002

* These variables were only taken into account for the records of telemedicine attention

Finally, 20 patients were referred to the emergency room from telemedicine consultation, mostly due to the presence of symptoms referred by the patient (80%), and the rest were referred due to alterations in laboratory tests (20%); 2.2% of the patients required hospitalization due to decompensation of the disease, and 29 (2.3%) deaths were recorded, without significant differences according to the area of residence (Table 4).

**Table 4:** Patient clinical outcomes by area of residence

Variables	Urban Area n = 704	Rural Area n = 566	Total n = 1270	p value
**Referral to the emergency room from the telemedicine attention**	10 (1.4%)	10 (1.8%)	20 (1.6%)	0.8
Patients reported symptoms^a^	8 (80.0%)	8 (80.0%)	16 (80.0%)	0.9
Alterations in lab test^a^	2 (20.0%)	2 (20.0%)	4 (20.0%)	
**Need for hospitalization**	7 (2.5%)	5 (1.9%)	12 (2.2%)	0.9
**Mortality**	21 (3.0%)	8 (1.4%)	29 (2.3%)	0.10

^a^ Percentages are in accordance with the number of patients referred to the emergency department for each group

## Discussion

### Principal findings

The principal solid tumor types were breast, prostate, and colon-rectum for both groups and the majority were in local status. There were no differences in physical exams and laboratory alterations. Those in the urban group were significantly more likely to be referred to other specialties than those in the rural area. In terms of the need for referral to the emergency department from telemedicine attention, the need for hospitalization, and deaths during the study period, there were no differences between the urban and rural groups.

### Results in context

Rurality is associated with higher incidence and mortality by cancer [13]; having a concentration of services in large cities, patients must travel long distances, imposing an additional geographical barrier to access, and since they are high-cost services, the profitability of taking them to neighboring municipalities is low [14]. Until now, videoconference tools have improved oncological service coverage to remote areas, with good patient and provider satisfaction, decreasing costs and distance of trips, and allowing higher access to oncologists [13, 15]. Telemedicine uses technology to offer remote health services and can be applied to screening, diagnosis, treatment, and follow-up practices [8, 16]. During the COVID-19 pandemic, telemedicine oriented to the care of oncological patients has expanded its adoption in multiple hospitals and settings to reduce exposure to the virus, especially in the oncology population, as they are more susceptible to severe manifestations and complications [16].

The solid tumors identified in our study are consistent with worldwide reports, with breast, prostate, lung, and colon and rectal cancers being the most prevalent [2] Breast and prostate cancer share the need for long-term oral or injectable management to avoid tumor relapse, either with endocrine therapy or hormonal blockade [17, 18]; this type of treatment allows greater autonomy and ease of management, which could explain why the telemedicine during the contingency was mainly composed of this type of tumor. In fact, international organizations such as the European Society for Medical Oncology (ESMO) recommended continuing with oral treatments whenever possible and the follow-up of survivors by telemedicine [19, 20].

Although the alterations in the physical examination were low, this aspect continues to be a concern and a challenge for telemedicine even when this section has been carried out by a local doctor or nurse in the rural area from where the connection between the patient and the specialist is made through telehealth strategies [15, 21, 22]. Walle et al. carried out a randomized clinical trial in which they sought to compare the feasibility of video consultations with in-person care for the management of patients with solid tumors who required follow-up of active treatment. They included a total of 66 subjects, randomized 1:1 in both groups. Although the differences in the interventions performed (referral to other specialties, physical examination, prescription of medications, and scheduling of control appointments) were not statistically significant, possibly due to the small sample size, the results tended to be lower compared with in-person care, mainly in performing a physical examination and prescription of medications [23].

For conducting a thorough physical examination during a videoconference evaluation [24]; the rapid implementation of this modality of care during the pandemic may result in a lack of training in physicians who are confronted with this type of evaluation for the first time. Furthermore, while it appears that telemedicine allows for good communication, it is critical to consider the training and learning of communication tools (body language, eye contact) that allow for better interaction with patients, which may be overlooked due to the emergent implementation of the modality. [25]. Specialists recognize the ability of telemedicine to anticipate visits to the emergency department or hospital admission, prevent nosocomial infections, or increase the frequency of medical visits [26].

On the other hand, we identified a minority of patients in whom some laboratory abnormalities were reported. As we have a larger population with their pathology in remission or maintenance treatment, this finding was expected. However, even though most of the subjects had stable diseases, it should be noted that a minority had metastatic disease or treatment with palliative intention, with palliative care being the most frequent specialty referral. Palliative care is essential in patients with metastatic disease, and the World Health Organization has declared this as a fundamental right of cancer patients [27], being a need recognized by specialists in the area. In our case, telemedicine permitted the identification of the patients’ needs and the referral to other important medical specialties in cancer follow-up, achieving the required multidisciplinary management.

During the COVID-19 pandemic, the ESMO issued a series of recommendations and guidelines for the management of cancer patients, categorizing them into priority levels based on the clinical status of the disease, indicating the use of telemedicine for those with medium and low priority in the medical oncologist’s opinion [28, 29]. These factors, which were used to select patients for telemedicine, may explain the low number of referrals to the emergency room and the low need for hospitalization in the two populations studied.

The literature demonstrates the satisfaction of patients, caregivers, and health personnel with oncology care through telemedicine, especially when it is offered in rural areas [30]. A study conducted in a rural population of Australia evaluated the satisfaction of patients and health personnel with regard to the videoconference tele-oncology program, revealing very positive results from both perspectives and demonstrating that it can be used for clinical follow-up as well as the inclusion and dosage of chemotherapy regimens. In addition to saving money on travel, telemedicine can be used as a supplement to care in any area of medicine for patients living in rural areas [15].

### Limitations and strengths

Our main limitation was the retrospective nature and short time period of the study. However, FVL is an institution that provides health care and is a reference center for the populations of the southwestern part of the country, where there is significant population and territorial heterogeneity that can be extrapolated to other territories of the country. To our knowledge, there are no similar studies in Colombia or Latin America to compare our results, so this study provides the basis for further large-scale studies to evaluate the behavior and economic impact of oncology patient care via telemedicine, strengthening this type of resource and encouraging better strategies to overcome the gaps in care for this population.

### Future implications

Rural populations face several limitations to access health care, not only in developing countries [31]. Previous experience in contexts other than Latin America suggests telehealth strategies to access cancer care among rural populations while providing quality of care and preserving low costs [32, 33]. This preliminary experience in a Latin American context, with similar results among urban and rural oncological cancer patients who were included in our study, supports the strengthening of these strategies to reduce access barriers and inequity.

## Conclusion

Telemedicine is a useful tool for follow-up by the oncology specialty in patients with solid tumors regardless of the area of residence since the outcomes are similar for both groups. Therefore, given its ease of installation and use, its adaptation should be promoted in low- and middle-income countries.

## Data Availability

The datasets analyzed during the current study and that support the findings of this study are available from Fundación Valle del Lili, but restrictions apply to the availability of these data due to internal privacy policies. Data are, however, available from Peña-Zárate E.E. (evelyn.pena@fvl.org.co) upon reasonable requests and with permission of Fundación Valle del Lili.
